# *Tinea capitis* in the Americas: Epidemiological Trends and Etiological Distribution from a Systematic Review

**DOI:** 10.3390/biology15161413

**Published:** 2026-08-17

**Authors:** Jessica Guzmán Pérez, Tania Vite-Garín, Rigoberto Hernández-Castro, Paola Berenice Zárate-Segura, Fernando Bastida-González, Roberto Arenas, José Pereira-Brunelli, Víctor de Jesús Suárez-Valencia, Rodolfo Pinto-Almazán, Erick Martínez-Herrera

**Affiliations:** 1 Sección de Estudios de Posgrado e Investigación, Escuela Superior de Medicina, Instituto Politécnico Nacional, Plan de San Luis y Díaz Mirón, Mexico City 11340, Mexico; jess.gupe@gmail.com (J.G.P.); tania.vite.garin@gmail.com (T.V.-G.); pbzars@yahoo.com (P.B.Z.-S.); 2 Facultad de Ciencias, Universidad Nacional Autónoma de México, Investigación Científica, C.U., Coyoacán, Mexico City 04510, Mexico; 3 Departamento de Biología Molecular e Histocompatibilidad, Hospital General Dr. Manuel Gea González, Tlalpan, Mexico City 14080, Mexico; rigo37@gmail.com; 4 Laboratorio de Biología Molecular, Laboratorio Estatal de Salud Pública del Estado de México, Toluca de Lerdo 50130, Mexico; mijomeil@hotmail.com; 5 Sección de Micología, Hospital General “Dr. Manuel Gea González”, Mexico City 14080, Mexico; rarenas98@hotmail.com; 6 Dermatological Specialties Center, Ministry of Public Health and Social Welfare, San Lorenzo 111419, Paraguay; jose_pereira15@hotmail.com; 7 Research Department, School of Medicine Saltillo Unit, Universidad Autónoma de Coahuila, Francisco Munguía Sur Esquina con Francisco I. Madero, Saltillo 25000, Mexico; victorsuarezvalencia@uadec.edu.mx; 8 Fundación Vithas, Grupo Hospitalario Vithas, 28043 Madrid, Spain

**Keywords:** *Tinea capitis*, *Microsporum canis*, *Trichophyton tonsurans*, epidemiological, Americas

## Abstract

Background: *Tinea capitis* remains one of the most common superficial fungal infections in children worldwide and represents an important public health concern due to its high prevalence, potential complications, and changing epidemiology. This study aimed to characterize the epidemiological trends and etiological distribution of *tinea capitis* across the Americas through a systematic review of the available literature. Methods: A systematic search was conducted in MEDLINE (PubMed), Scopus, SCILIT, and SciELO following PRISMA guidelines. A total of 250 records were identified, of which 75 duplicates were removed. After title and abstract screening and eligibility assessment, 90 studies were included. Observational studies, prevalence studies, case series, and case reports reporting epidemiological or etiological data on *tinea capitis* in countries of the American continent were considered eligible. Methodological quality was assessed using the Joanna Briggs Institute (JBI) critical appraisal tools. No formal certainty-of-evidence assessment or meta-analysis was performed due to the descriptive nature and heterogeneity of the included studies. This review was not prospectively registered. Results: The 90 included studies comprised 506,086 reported cases of *tinea capitis*. Marked regional differences were identified in the distribution of dermatophyte species. *Trichophyton tonsurans* predominated in North America and several Caribbean countries, whereas *Microsporum canis* was the most frequently reported etiological agent in much of South America and parts of Central America, suggesting an important role of zoonotic transmission in these regions. Variations in prevalence and species distribution were also associated with geographic, socioeconomic, and demographic factors. Overall, the included studies demonstrated low-to-moderate methodological risk of bias. Conclusions: These findings demonstrate substantial heterogeneity in the epidemiology of tinea *capitis* throughout the Americas and highlight the importance of continuous surveillance, accurate mycological diagnosis, and region-specific public health strategies to improve disease control and guide appropriate therapeutic interventions.

## Simple Summary

*Tinea capitis* is a fungal infection of the scalp and hair shafts that primarily affects children and remains one of the most common superficial mycoses worldwide. Although the disease is generally treatable, delayed diagnosis and inadequate management may lead to inflammatory complications, permanent scarring alopecia, and continued transmission within households, schools, and other community settings. The epidemiology of *tinea capitis* is highly dynamic and varies substantially according to geographic region, socioeconomic conditions, migration patterns, urbanization, and contact with domestic animals. This systematic review provides a comprehensive overview of the epidemiological characteristics and etiological distribution of *tinea capitis* across the Americas. Studies conducted in North America, Central America, the Caribbean, and South America were analyzed to identify the predominant dermatophyte species and regional trends. The findings demonstrate significant geographic variation in causative agents. *Trichophyton tonsurans* was the predominant pathogen in North America and several Caribbean countries, whereas *Microsporum canis* remained the leading etiological agent in much of South America and parts of Central America, reflecting the importance of zoonotic transmission in these regions. By summarizing the available evidence from across the continent, this review highlights changing epidemiological patterns and underscores the need for continuous surveillance, accurate mycological diagnosis, and region-specific public health strategies to improve disease control and optimize patient management.

## 1. Introduction

*Tinea capitis* is a dermatophytic infection of the scalp and hair shaft and remains one of the most prevalent superficial mycoses among children worldwide. Its clinical presentation ranges from mild scaling, often resembling seborrheic dermatitis, to severe inflammatory forms such as kerion, which may lead to permanent cicatricial alopecia if not promptly treated [1,2]. In addition, subclinical infections play an important role in sustaining transmission within susceptible populations through asymptomatic carriers and close interpersonal contact.

From an etiological perspective, *tinea capitis* is mainly caused by dermatophytes of the genera *Trichophyton* and *Microsporum*, whose distribution varies considerably according to geographic region [3]. In the Americas, the disease represents a substantial proportion of pediatric dermatophytoses, accounting for up to 44% of reported cases in children, particularly among preschool and school-aged populations [3,4]. Transmission occurs primarily through direct contact with infected individuals or indirectly via contaminated fomites such as combs, brushes, and personal objects, with frequent intrafamilial spread and clustering in school settings [1,2]. Socioeconomic factors, including overcrowding and poor hygiene conditions, have also been recognized as critical determinants favoring disease dissemination [3].

The epidemiology of *tinea capitis* is highly dynamic and influenced by demographic, environmental, and social factors. Although *Microsporum canis* has historically been the predominant etiological agent in several Latin American countries, recent reports have documented the increasing emergence of anthropophilic species such as *Trichophyton tonsurans*, particularly in urban populations and migratory contexts [3]. These epidemiological shifts highlight the importance of continuous surveillance to better understand regional trends and transmission patterns across the continent.

From a biological standpoint, the increased susceptibility observed in children has been associated with the composition of the prepubertal cutaneous mycobiome [5]. Furthermore, advances in molecular methodologies have enabled more accurate identification of dermatophyte species and improved understanding of the phylogenetic dynamics underlying these infections.

In this context, the marked variability in etiological agents and prevalence patterns across the American continent underscores the need for an updated and systematic synthesis of the available evidence. Despite its epidemiological relevance, currently available data remain heterogeneous regarding study design, population characteristics, and diagnostic approaches, limiting comparability among studies and hindering a comprehensive understanding of disease behavior in the region. Therefore, this study aims to systematically review the literature on the epidemiology, prevalence, and causative agents of *tinea capitis* in the Americas in order to integrate current evidence and provide an updated perspective to inform future research and control strategies. We hypothesized that substantial regional differences exist in the etiological distribution of *tinea capitis* across the Americas.

## 2. Materials and Methods

### 2.1. Study Selection and Eligibility Criteria

A systematic literature search was conducted using the MEDLINE (PubMed) https://pubmed.ncbi.nlm.nih.gov/ (accessed on 17 March 2026), Scopus https://www.scopus.com/ (accessed on 19 March 2026), SCILIT https://www.scilit.com/ (accessed on 18 March 2026) and SciELO https://www.scielo.org/es/ (accessed on 17 March 2026) databases to identify studies published between 2000 and 2025 addressing the epidemiology and etiological distribution of *tinea capitis* in the American continent. The following search terms were used: “*Tinea capitis*”, “Dermatophyte”, “*Trichophyton*”, “*Microsporum*”, “*Nannizzia*”, “America”, “North America”, “Central America”, “South America”, “Caribbean”, as well as the names of individual countries within each geographic region of the continent. The final search was performed on 15 March 2026 and included studies published through December 2025. No review protocol was prepared. This review was not prospectively registered.

Case reports and small case series were intentionally included because the primary objective of this systematic review was to comprehensively characterize the etiological diversity and geographic distribution of *tinea capitis* across the Americas rather than to estimate pooled prevalence or incidence. Although these studies contribute limited epidemiological weight and were not considered representative of regional species predominance, they provide valuable evidence of uncommon, emerging, or geographically restricted dermatophyte species that may otherwise be overlooked. Their inclusion therefore contributes to a more comprehensive understanding of the current etiological landscape of *tinea capitis* in the Americas.

For the purposes of narrative synthesis, greater emphasis was placed on findings from epidemiological studies with larger sample sizes when describing regional patterns, whereas isolated case reports were interpreted as complementary evidence of etiological diversity.

Search Strategy

The literature search strategy was developed to identify studies reporting epidemiological, prevalence, or etiological data on *tinea capitis* in countries of the American continent. Searches were conducted in MEDLINE (PubMed), Scopus, SCILIT, and SciELO using combinations of controlled vocabulary terms and free-text keywords related to *tinea capitis*, dermatophytes, etiological agents, and geographic regions.

The primary search strategy included the following terms and Boolean operators:

(“*tinea capitis*” OR dermatophyte* OR *Trichophyton* OR *Microsporum* OR *Nannizzia*) AND (“America” OR “North America” OR “Central America” OR “South America” OR Caribbean OR Canada OR “United States,” OR Mexico OR Guatemala OR Cuba OR Haiti OR Jamaica OR “Dominican Republic” OR Costa Rica OR Argentina OR Bolivia OR Brazil OR Chile OR Colombia OR Ecuador OR “French Guiana” OR Paraguay OR Peru OR Uruguay OR Venezuela)

Searches were limited to studies published between January 2000 and March 2025 in English, Spanish, or Portuguese. The reference lists of all eligible articles were manually screened to identify additional relevant studies not retrieved through database searching. The complete search strategies for each database are provided in Supplementary Table S1.

**Figure 1:**
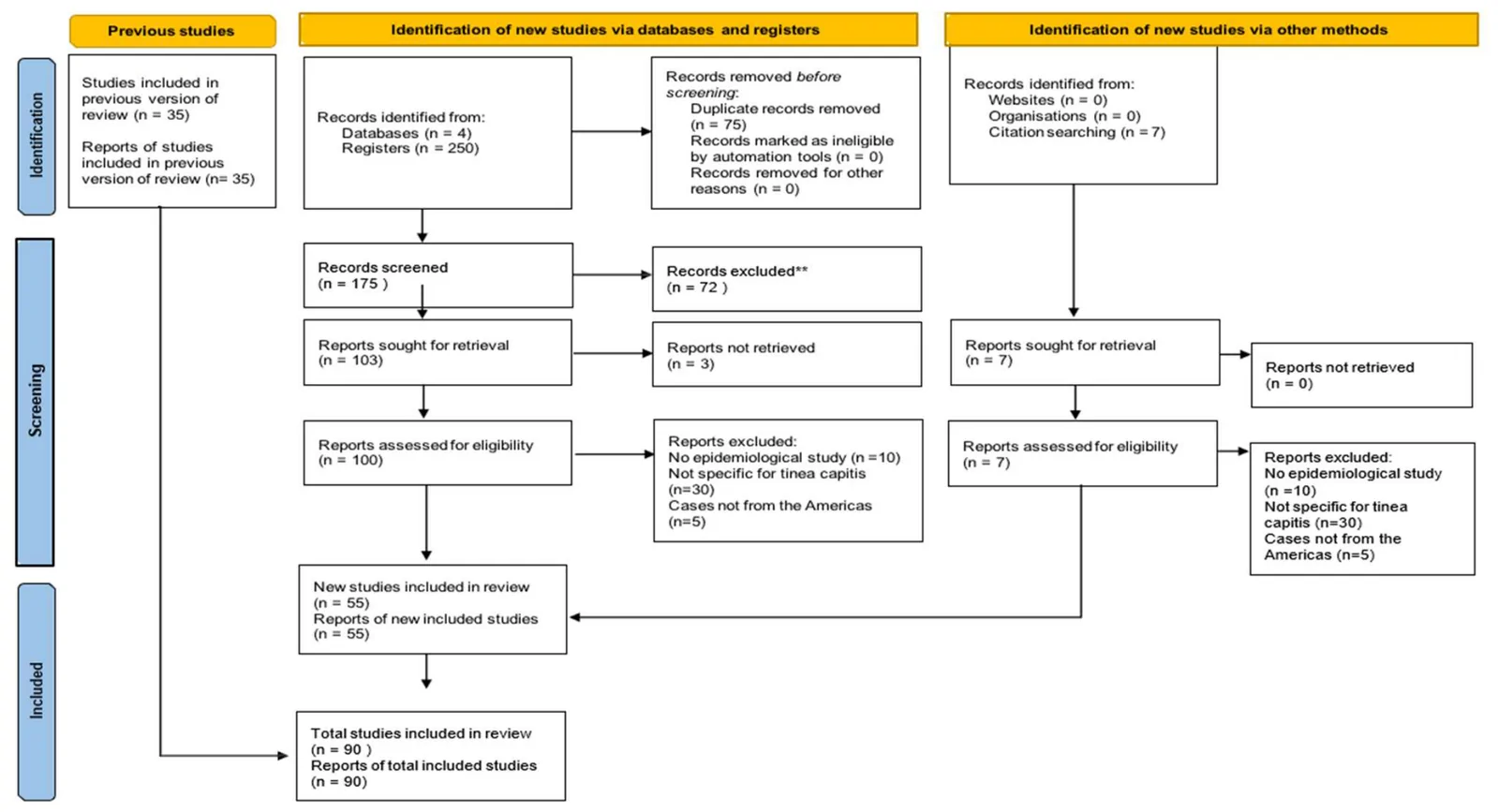
PRISMA 2020 flow diagram for updated systematic reviews, which included searches of databases, registers and other sources. **: If automation tools were used, indicate how many records were excluded by a human and how many were excluded by automation tools.

A PRISMA flow diagram was constructed to document the identification, screening, eligibility assessment, and inclusion process, ensuring a systematic and transparent study selection methodology (Figure 1). A total of 250 records were initially identified through database searching. After the removal of 75 duplicate records, the remaining studies underwent title and abstract screening. Articles published between 2000 and 2025 in English, Spanish, or Portuguese were included, comprising prevalence studies, cross-sectional studies, retrospective observational studies, case series, and case reports [6].

#### 2.1.1. Data Acquisition

Study selection, title and abstract screening, eligibility assessment, and data extraction were independently performed by Guzmán-Pérez and Vite-Garín. Any discrepancies were resolved by consensus between both reviewers. The reference lists of all selected articles were manually reviewed to identify additional potentially relevant studies. The exclusion criteria included: unspecified etiological genus or species, lack of epidemiological relevance to *tinea capitis*, insufficient clinical or microbiological information, unavailable abstract or full text, duplicate publications, conference abstracts lacking complete data, studies focused exclusively on pharmacological treatment, and experimental studies based solely on molecular techniques, gene expression analyses, or cell culture methods without associated epidemiological or clinical correlation.

Eligibility for data extraction was determined according to the availability of *tinea capitis*-specific epidemiological and etiological information rather than the primary focus reflected in the publication title. Consequently, studies addressing broader dermatological or mycological conditions were included when they reported extractable data specific to tinea capitis. Each eligible study was independently reviewed in full to ensure that only relevant information was incorporated into the qualitative synthesis.

Data extraction was conducted using a predefined Microsoft Excel spreadsheet. Outcomes were reported individually for each study and included geographic region, country, city or study area, number of *tinea capitis* cases, predominant sex, age, age group, etiological agent, diagnostic method, and bibliographic reference. When data were unavailable or not specified in the original publication, they were recorded as NA (not available). Age groups were classified according to the World Health Organization (WHO) age classification system, including neonate, infant, child, adolescent, young adult, adult, older adult, senior, and oldest-old [7,8].

The included studies were subsequently organized according to the geographic regions of the American continent: North America, Central America and the Caribbean, and South America, in order to facilitate comparative analysis of epidemiological patterns and etiological distribution across the continent.

#### 2.1.2. Quality Assessment

The methodological quality of the included studies was assessed using the Joanna Briggs Institute (JBI) critical appraisal tools, selected according to the corresponding methodological design of each publication, including prevalence studies, observational studies, and case reports.

- *Risk of bias in individual studies*

Two reviewers (Guzman-Perez and Vite-Garin) jointly appraised the included studies using the Joanna Briggs Institute (JBI) critical appraisal tools according to the methodological design of each publication, including prevalence studies, observational studies, case series, and case reports. Each checklist item was rated according to JBI guidance, and studies were not excluded based on quality scores. The assessment was used to evaluate methodological rigor and to support interpretation of the findings.

Studies scoring ≥70% of the applicable JBI items were classified as low risk of bias, scores between 50–69% as moderate risk, and scores <50% as high risk. (Table S1).

b. *Risk of bias between studies*

A formal quantitative assessment of publication bias was not performed because the review did not include a meta-analysis and the included studies were highly heterogeneous. To minimize potential bias, a comprehensive search strategy was applied across multiple databases, and reference lists were manually reviewed to identify additional eligible studies.

It is important to note that in this review, genera and species are cited exactly as they appear in the reviewed articles, despite the new taxonomic nomenclature and classification under which some of them have been re-classified and renamed.

c. *Synthesis methods*

A qualitative narrative synthesis was performed to summarize the epidemiological characteristics and etiological distribution of *tinea capitis* across the Americas. Given the substantial heterogeneity among included studies regarding study design, population characteristics, geographic regions, diagnostic methods, and outcome reporting, quantitative pooling of data was not considered appropriate. Therefore, findings were synthesized descriptively and organized according to geographic region (North America, Central America and the Caribbean, and South America) to facilitate comparison of epidemiological patterns and causative dermatophyte species.

d. *Sensitivity analyses*

Sensitivity analyses were not performed because no quantitative synthesis or meta-analysis was conducted. The included studies exhibited considerable methodological and clinical heterogeneity, precluding formal statistical evaluation of the robustness of pooled estimates.

e. *Certainty of Evidence Assessment*

A formal certainty of evidence assessment using GRADE was not performed because this systematic review was primarily descriptive and did not include quantitative effect estimates, comparative interventions, or meta-analytic synthesis. The included studies were highly heterogeneous in terms of study design, population characteristics, diagnostic methods, geographic setting, and outcome reporting, which limited the applicability of a formal GRADE assessment. Therefore, the certainty of the evidence was interpreted narratively, considering methodological quality, consistency of findings across regions, diagnostic heterogeneity, and completeness of reporting.

### 2.2. Protocol Registration

This systematic review was not prospectively registered in PROSPERO or any other international registry. At the time the review was initiated, protocol registration had not been performed. Although prospective registration is recommended to enhance methodological transparency, the review was conducted following the PRISMA 2020 statement, and all eligibility criteria, search strategies, data extraction procedures, and risk-of-bias assessments were predefined and consistently applied throughout the review process.

## 3. Results

### 3.1. General Characteristics of the Included Studies

The 90 studies included in the present systematic review comprised a total of 506,086 reported cases of *tinea capitis* across the American continent, encompassing North America, Central America and the Caribbean, and South America. The largest proportion of publications originated from South America, followed by North America and Central America/the Caribbean. Brazil, Mexico, and the United States accounted for the highest number of reported studies (Table 1 and Table 2) [1,2,3,4,5,9,10,11,12,13,14,15,16,17,18,19,20,21,22,23,24,25,26,27,28,29,30,31,32,33,34,35,36,37,38,39,40,41,42,43,44,45,46,47,48,49,50,51,52,53,54,55,56,57,58,59,60,61,62,63,64,65,66,67,68,69,70,71,72,73,74,75,76,77,78,79,80,81,82,83,84,85,86,87].

Overall, the included studies predominantly involved pediatric populations. Children and adolescents represented the most frequently affected age groups, whereas adults and older individuals accounted for a substantially smaller proportion of cases. Male predominance was observed in the majority of the included studies [1,2,3,4,5,6,7,8,9,10,11,12,13,14,15,16,17,18,19,20,21,22,23,24,25,26,27,28,29,30,31,32,33,34,35,36,37,38,39,40,41,42,43,44,45,46,47,48,49,50,51,52,53,54,55,56,57,58,59,60,61,62,63,64,65,66,67,68,69,70,71,72,73,74,75,76,77,78,79,80,81,82,83,84,85,86,87].

Regarding etiological distribution, *M. canis* and *T. tonsurans* were identified as the predominant causative agents throughout the continent. Nevertheless, substantial regional epidemiological differences were observed [1,2,3,4,5,7,8,9,10,11,12,13,14,15,16,17,18,19,20,21,22,23,24,25,26,27,28,29,30,31,32,33,34,35,36,37,38,39,40,41,42,43,44,45,46,47,48,49,50,51,52,53,54,55,56,57,58,59,60,61,62,63,64,65,66,67,68,69,70,71,72,73,74,75,76,77,78,79,80,81,82,83,84,85,86,87].

With respect to diagnostic approaches, the combination of direct microscopic examination using potassium hydroxide (KOH) and fungal culture represented the most commonly employed diagnostic strategy across the included studies. A smaller number of publications relied exclusively on direct microscopy, fungal culture alone, or complementary histopathological methods [1,2,3,4,5,7,8,9,10,11,12,13,14,15,16,17,18,19,20,21,22,23,24,25,26,27,28,29,30,31,32,33,34,35,36,37,38,39,40,41,42,43,44,45,46,47,48,49,50,51,52,53,54,55,56,57,58,59,60,61,62,63,64,65,66,67,68,69,70,71,72,73,74,75,76,77,78,79,80,81,82,83,84,85,86,87]

To facilitate interpretation of the qualitative synthesis, the principal epidemiological trends and etiological patterns identified across the Americas are summarized in Table 1. This regional overview highlights the predominant dermatophyte species, the most frequently reported secondary species, the prevailing transmission patterns, and the main epidemiological trends observed within each geographic region. Despite substantial methodological heterogeneity among the included studies, consistent regional differences in dermatophyte distribution were identified throughout the continent.

### 3.2. North America

North America represented one of the regions with the highest number of included studies, comprising investigations conducted primarily in Canada, the United States, and Mexico [9,10,11,12,13,14,15,16,17,18,19,20,21,22,23,24,25,26,27,28,29,30,31,32,33]. Marked epidemiological and etiological heterogeneity was observed across the region, with notable differences among countries and study populations.

In the United States, *T. tonsurans* was consistently reported as the predominant etiological agent in most studies, particularly among pediatric populations. In addition, emerging anthropophilic species such as *Trichophyton violaceum* and *Trichophyton soudanense* were identified, especially in metropolitan areas characterized by high population diversity and migration-related demographic changes [13,14,15,16,17,18,19,20,21,22,23].

Similarly, anthropophilic dermatophytes predominated in Canada. Studies conducted in Montreal reported high frequencies of *Microsporum audouinii*, *T. soudanense*, and *T. violaceum*, whereas investigations from Toronto documented a predominance of *M. canis* and *T. tonsurans*. These findings suggest a substantial influence of migration patterns and demographic shifts on the regional epidemiology of *tinea capitis* [9,10,11,12].

In contrast, Mexico exhibited a predominantly zoophilic epidemiological profile, with *M. canis* representing the leading etiological agent in multiple studies. Nevertheless, several investigations also documented the presence of *T. tonsurans*, *Trichophyton rubrum*, *Nannizzia gypsea*, and *Nannizzia nana*, reflecting a more heterogeneous etiological distribution in specific geographic areas [24,25,26,27,28,29,30,31,32,33].

Pediatric populations, particularly school-aged children and adolescents, represented the most frequently affected age groups throughout North America (Table 1). Male predominance was consistently observed across most studies. Regarding diagnostic practices, the combination of direct microscopy and fungal culture constituted the most frequently utilized diagnostic approach in the region [9,10,11,12,13,14,15,16,17,18,19,20,21,22,23,24,25,26,27,28,29,30,31,32,33].

### 3.3. Central America and the Caribbean

Central America and the Caribbean exhibited considerable epidemiological diversity in the etiological agents associated with *tinea capitis*, with coexistence of both anthropophilic and zoophilic dermatophytes. The included studies originated primarily from Guatemala, Costa Rica, Cuba, the Dominican Republic, Haiti, and Jamaica [1,35,36,37,38,39,40,41,42,43,44].

In Guatemala, *M. canis* predominated in most epidemiological series, reaching frequencies exceeding 80% in some studies. However, isolates of *T. rubrum*, *T. mentagrophytes*, *M. gypseum*, and *M. audouinii* were also reported, indicating a relatively heterogeneous etiological distribution [34,35,36].

Conversely, several studies from Caribbean countries documented a marked predominance of anthropophilic species, particularly T. tonsurans. In the Dominican Republic, Haiti, and Jamaica, *T. tonsurans* was among the most frequently isolated etiological agents, especially in pediatric populations [38,39,40,41,42]. Other relevant anthropophilic species identified included *M. audouinii* and *T. rubrum* [35,36,39,41,42,43].

Certain countries within the region reported sporadic cases involving less frequently encountered species. In Costa Rica, cases associated with *T. mentagrophytes* and *M. gypseum* were documented, whereas Cuba reported an isolated case caused by *M. canis*. These findings highlight the persistence of zoophilic dermatophytes in selected geographic areas of Central America and the Caribbean [1,37,38].

Pediatric populations, particularly school-aged children, represented the most frequently affected demographic group throughout the region. Similar to other regions of the continent, male predominance was observed in most studies. Direct microscopy combined with fungal culture constituted the most widely employed diagnostic approach for etiological identification [1,34,35,36,37,38,39,40,41,42,43].

### 3.4. South America

South America represented the region with the largest number of included studies in the present systematic review, encompassing investigations conducted in Argentina, Brazil, Bolivia, Chile, Colombia, Ecuador, French Guiana, Paraguay, Peru, Uruguay, and Venezuela. Overall, the region demonstrated a clear predominance of zoophilic dermatophytes, particularly *M. canis*, although significant epidemiological variability was observed among countries [5,44,45,46,47,48,49,50,51,52,53,54,55,56,57,58,59,60,61,62,63,64,65,66,67,68,69,70,71,72,73,74,75,76,77,78,79,80,81,82,83,84,85,86].

Argentina contributed one of the largest bodies of epidemiological evidence in the region. Most studies identified M. canis as the predominant etiological agent, with frequencies exceeding 80% in several pediatric series [5,44,45,46,47,48,49,50]. Nevertheless, the presence of *T. tonsurans*, *T. mentagrophytes*, *M. gypseum*, and *T. rubrum* was also documented, indicating a relatively diverse etiological landscape [5,44,45,46,48,49,50,51,52,53,54,55,57,58,59,60,61,62,65,66,67,68,70,72,73,75,76,77,78,79,80,81,82,83,84,85,86,87].

Brazil accounted for the highest number of included publications and exhibited substantial regional epidemiological heterogeneity. Although *M. canis* predominated in numerous studies, certain regions reported a higher frequency of *T. tonsurans*. Additional etiological agents identified included *T. rubrum*, *T. mentagrophytes*, *N. gypsea*, and *T. violaceum*. These findings suggest a complex interaction among geographic, climatic, demographic, and socioeconomic factors influencing the epidemiology of *tinea capitis* in Brazil [50,51,52,53,54,55,56,57,58,59,60,61,62,63,64,65,66,67,68,69].

In Chile, Colombia, Peru, and Venezuela, *M. canis* overwhelmingly predominated, particularly among pediatric populations. Several studies reported high frequencies of this species, although occasional isolates of *T. tonsurans*, *T. rubrum*, and *M. gypseum* were also documented [2,4,71,72,73,74,75,76,77,83,84,86,87].

Paraguay displayed a particularly heterogeneous epidemiological pattern, with substantial frequencies of both *M. canis* and *T. mentagrophytes*, in addition to isolates of *T. tonsurans*, *T. rubrum*, and *M. gypseum* [79,80,81,82]. Similarly, studies from Ecuador and Bolivia reported diverse etiological profiles, including both anthropophilic and zoophilic species [70,78].

Pediatric populations constituted the predominantly affected age group throughout South America, particularly school-aged children and adolescents. Male predominance was reported in most studies. Regarding diagnostic practices, direct microscopy combined with fungal culture represented the most frequently employed diagnostic approach, whereas a smaller number of studies relied exclusively on direct microscopy or complementary methods such as skin biopsy [5,44,45,46,47,48,49,50,51,52,53,54,55,56,57,58,59,60,61,62,63,64,65,66,67,68,69,70,71,72,73,74,75,76,77,78,79,80,81,82,83,84,85,86].

### 3.5. Certainty of Evidence

Because no formal GRADE assessment was performed, the certainty of evidence was interpreted narratively. Overall, the evidence was considered descriptive and heterogeneous. The most consistent findings were the predominance of pediatric cases, the frequent use of direct microscopy and fungal culture, and the regional variation in etiological agents, particularly the predominance of *M. canis* in much of South America and *T. tonsurans* in North America and several Caribbean countries. However, confidence in regional comparisons is limited by heterogeneity in study design, diagnostic methods, reporting quality, and geographic representation.

## 4. Discussion

**Figure 2:**
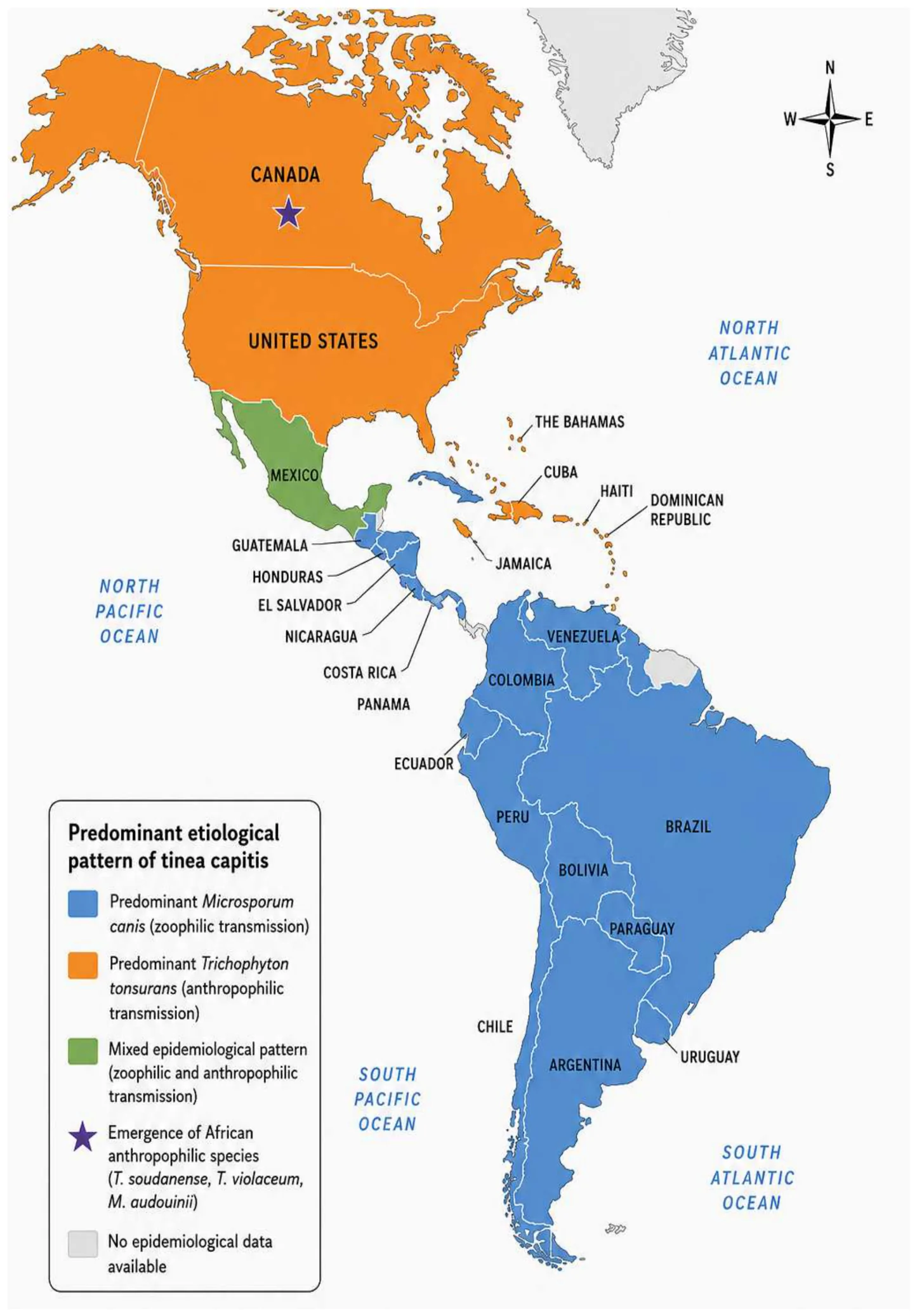
Geographic distribution of the predominant epidemiological patterns of *tinea capitis* across the Americas. Blue indicates regions where *M. canis* is the predominant etiological agent, orange indicates predominance of *T. tonsurans*, green indicates mixed epidemiological patterns, and the purple star denotes regions reporting the emergence of traditionally African anthropophilic dermatophytes (*T. soudanense*, *T. violaceum*, and *M. audouinii*).

This systematic review demonstrates that the epidemiology of *tinea capitis* in the Americas is characterized by marked geographic and etiological heterogeneity (Figure 2). Although the disease remains predominantly a pediatric infection throughout the continent, important regional differences were identified in the distribution of dermatophyte species, reflecting the complex interaction of environmental, socioeconomic, demographic, cultural, and ecological factors that influence fungal transmission. Collectively, these findings emphasize that *tinea capitis* should not be considered a homogeneous disease across the Americas, but rather a dynamic infectious entity whose epidemiology varies according to local transmission patterns and population characteristics.

Beyond the regional distribution identified in this review, the available literature also suggests important temporal changes in the epidemiology of *tinea capitis* throughout the Americas. Earlier studies from several Latin American countries consistently reported *M. canis* as the predominant etiological agent, reflecting predominantly zoonotic transmission. More recent publications, however, describe an increasing frequency of anthropophilic species, particularly *T. tonsurans*, in urban settings and highly populated regions. Although the heterogeneity of study designs, study periods, and reporting methods precluded a formal temporal trend analysis, the collective evidence suggests a progressive epidemiological shift likely driven by urbanization, migration, demographic changes, globalization, and evolving patterns of human-to-human transmission. These findings support the concept that the epidemiology of dermatophytosis is continuously evolving and requires ongoing surveillance to detect emerging changes in species distribution [2,4,5,24,25,26,27,28,29,30,31,32,33,34,35,36,44,45,46,47,48,49,50,51,74,75,76,77,78,79,80,87,88].

One of the most relevant findings of this review was the clear distinction between regions where zoophilic dermatophytes remain predominant and those where anthropophilic species have become established. *M. canis* remained the leading etiological agent throughout much of South America, particularly in Argentina, Chile, Colombia, Peru, and Venezuela, as well as in several regions of Mexico and Guatemala, reinforcing the continued importance of zoonotic transmission associated with close contact with domestic animals, particularly cats and dogs [2,4,5,24,25,26,27,28,29,30,31,32,33,34,35,36,44,45,46,47,48,49,50,51,73,74,75,76,77,78,79,86,87,90,91]. Conversely, *T. tonsurans* predominated in the United States and several Caribbean countries, confirming its role as the principal anthropophilic dermatophyte in these populations. Similar epidemiological patterns have been described in other highly urbanized regions worldwide, where transmission occurs primarily through close interpersonal contact within households, schools, daycare centers, and recreational facilities [9,10,11,12,13,14,15,16,17,18,19,20,21,22,23,24,25,26,27,28,29,30,31,32,33,39,40,41,42,43].

The emergence of traditionally African dermatophyte species, including *T. soudanense*, *T. violaceum*, and *M. audouinii*, particularly in Canada and other metropolitan areas, deserves special attention. Increasing international migration, globalization, international travel, and demographic diversification have likely contributed to the introduction and subsequent establishment of these species outside their historical endemic regions. Comparable epidemiological shifts have been reported throughout Europe, suggesting that global population mobility is progressively modifying the geographic distribution of dermatophytes. Continuous epidemiological surveillance will therefore become increasingly important for early recognition of newly emerging species and changing transmission patterns [9,10,11,12].

From a clinical perspective, the regional differences identified in this review have important practical implications. Knowledge of the predominant dermatophyte species within a particular geographic area may facilitate more appropriate empirical therapeutic decisions while mycological confirmation is pending. In regions where *T. tonsurans* predominate, clinicians should anticipate predominantly anthropophilic transmission and reinforce household screening, school-based infection control measures, and early identification of asymptomatic carriers. Conversely, in regions where *M. canis* remains the principal etiological agent, investigation of animal reservoirs, veterinary evaluation of household pets, and interventions aimed at reducing zoonotic transmission should constitute integral components of disease management. These epidemiological differences may also support the development of region-specific diagnostic algorithms and public health strategies adapted to local epidemiological conditions [4,5,10,11,14,15,16,18,19,20,21,24,25,26,28,29,30,31,33,34,35,36,38,39,40,41,42,43,44,45,46,47,48,49,50,51,52,53,54,55,56,57,58,59,60,61,62,63,65,66,67,68,69,70,72,73,74,75,76,77,78,79,80,81,82,83,84,85,86,87,88,89,90].

Consistent with previous international studies, children and adolescents represented the most frequently affected population across all regions included in this review. Several biological and behavioral factors probably contribute to this distribution, including the reduced fungistatic activity of prepubertal sebum, increased close interpersonal contact, and greater exposure to infected individuals, fomites, and domestic animals. Likewise, the predominance of male patients observed in most studies is consistent with previous reports, although the biological and behavioral mechanisms responsible for this difference remain incompletely understood [1,2,3,4,5,9,10,11,12,13,14,15,16,17,18,19,20,21,22,23,24,25,26,27,28,29,30,31,32,33,34,35,36,37,38,39,40,41,42,43,44,45,46,47,48,49,50,51,52,53,54,55,56,57,58,59,60,61,62,63,64,65,66,67,68,69,70,71,72,73,74,75,76,77,78,79,80,81,82,83,84,85,86,87,88,89,90,91].

Direct microscopic examination combined with fungal culture remained the most frequently employed diagnostic approach throughout the continent and continues to represent the reference standard in most epidemiological investigations. Nevertheless, one of the principal limitations identified in the available literature was the considerable heterogeneity of diagnostic methodologies. Differences in laboratory expertise, specimen collection techniques, culture media, incubation conditions, identification criteria, and taxonomic updates may have substantially influenced the reported distribution of dermatophyte species. Furthermore, the gradual incorporation of molecular diagnostic methods in recent years has likely improved species identification in more recent publications compared with older studies relying exclusively on phenotypic characterization. Consequently, part of the observed geographic variability may reflect methodological differences rather than true epidemiological variation, and this limitation should be considered when interpreting comparisons among countries and regions.

The growing implementation of molecular diagnostic techniques, including polymerase chain reaction (PCR)-based assays, sequencing of the internal transcribed spacer (ITS) region, matrix-assisted laser desorption/ionization time-of-flight mass spectrometry (MALDI-TOF MS), and other molecular approaches, is expected to substantially improve future epidemiological surveillance. These methodologies provide more accurate species identification, facilitate recognition of cryptic or emerging dermatophyte species, and allow better characterization of transmission dynamics than conventional phenotypic methods alone. Wider adoption of these techniques may therefore contribute to a more precise understanding of changing epidemiological patterns throughout the Americas [1,2,3,4,5,9,10,11,12,13,14,15,16,17,18,19,20,21,22,23,24,25,26,27,28,29,30,31,32,33,34,35,36,37,38,39,40,41,42,43,44,45,46,47,48,49,50,51,52,53,54,55,56,57,58,59,60,61,62,63,64,65,66,67,68,69,70,71,72,73,74,75,76,77,78,79,80,81,82,83,84,85,86,87,88,89,90,91].

Despite the broad geographic coverage and large number of included cases, this review has several limitations. The available evidence was derived predominantly from observational studies, prevalence studies, case series, and case reports that exhibited considerable methodological heterogeneity, variable reporting quality, and differences in diagnostic approaches. In addition, epidemiological information remains limited or unavailable for several countries within the American continent, potentially restricting the representativeness of regional estimates [1,2,3,4,5,9,10,11,12,13,14,15,16,17,18,19,20,21,22,23,24,25,26,27,28,29,30,31,32,33,34,35,36,37,38,39,40,41,42,43,44,45,46,47,48,49,50,51,52,53,54,55,56,57,58,59,60,61,62,63,64,65,66,67,68,69,70,71,72,73,74,75,76,77,78,79,80,81,82,83,84,85,86,87,88,89,90,91].

Although a formal methodological quality assessment demonstrated that most included studies presented low-to-moderate risk of bias, the descriptive nature of the available evidence and the absence of standardized diagnostic methodologies limited direct comparisons among studies.

Furthermore, the substantial heterogeneity of study designs and outcome reporting precluded quantitative synthesis and formal meta-analysis. Nevertheless, the consistency of the principal epidemiological findings across multiple geographic regions supports the overall robustness of the conclusions presented.

Overall, this review provides a comprehensive synthesis of the current epidemiological landscape of *tinea capitis* throughout the Americas and demonstrates that dermatophyte distribution continues to evolve in response to demographic, environmental, and societal changes.

Continued epidemiological surveillance using standardized diagnostic methodologies and broader implementation of molecular identification techniques will be essential to monitor future changes in species distribution, improve diagnostic accuracy, optimize empirical therapeutic strategies, and guide region-specific public health interventions.

## 5. Conclusions

*Tinea capitis* remains an important superficial fungal infection throughout the Americas, predominantly affecting pediatric populations. This systematic review demonstrates marked epidemiological heterogeneity across the continent, with *M. canis* predominating in most of South America and parts of Mexico and Central America, whereas *T. tonsurans* is the principal etiological agent in the United States and several Caribbean countries. The increasing detection of traditionally African anthropophilic dermatophytes in North America further suggests that migration and demographic changes are contributing to the evolving epidemiology of *tinea capitis*.

These findings highlight the importance of continuous epidemiological surveillance and region-specific diagnostic and therapeutic strategies. Future studies using standardized diagnostic methodologies and broader geographic representation are needed to improve our understanding of temporal trends and emerging etiological patterns of *tinea capitis* across the Americas.

## 6. Limitations

Methodological limitations of this review include heterogeneity among study designs, regional variations in diagnostic methods, differences in microbiological criteria for etiological identification, and the limited availability of epidemiological data from certain countries within the American continent. Additionally, publication bias cannot be completely excluded, as studies reporting uncommon etiological agents or negative findings may be underrepresented in the literature. The predominance of retrospective observational studies and variability in reporting quality may also have influenced the comparability of epidemiological estimates across regions.

## Figures and Tables

**Table 1:** Regional epidemiological and etiological patterns of *tinea capitis* in the Americas.

**Region**	**Predominant Dermatophyte Species**	**Frequently Reported Secondary Species**	**Predominant Transmission Pattern**	**Observed Epidemiological Trend**
North America	*Trichophyton tonsurans*	*Microsporum canis*, *T. soudanense*, *T. violaceum*, *M. audouinii*	Anthropophilic	Predominance of anthropophilic transmission, with increasing reports of traditionally African species, particularly in Canada, likely associated with migration and demographic changes.
Mexico	*Microsporum canis*	*T. tonsurans*, *T. rubrum*, *T. mentagrophytes*	Mixed (predominantly zoophilic)	Regional variability was observed, with *M. canis* remaining the predominant species in most studies, while *T. tonsurans* has become increasingly reported in urban populations.
Central America	*Microsporum canis*	*M. gypseum*, *T. tonsurans*, *T. mentagrophytes*, *T. rubrum*	Predominantly zoophilic	A relatively stable epidemiological pattern characterized by zoonotic transmission, with *M. canis* remaining the principal etiological agent.
Caribbean	*Trichophyton tonsurans*	*M. canis*, *M. audouinii*, *T. mentagrophytes*	Anthropophilic	Persistent predominance of anthropophilic transmission, particularly among school-aged children, with sustained circulation of *T. tonsurans* across multiple islands.
South America	*Microsporum canis*	*T. tonsurans*, *T. mentagrophytes*, *T. rubrum*, *M. gypseum*	Predominantly zoophilic	*M. canis* remains the predominant dermatophyte throughout most countries, although localized increases in anthropophilic species, particularly *T. tonsurans*, have been reported in some urban centers.

**Table 2:** Complete characteristics of included studies evaluating *Tinea capitis* in the Americas.

Region	Country	City/Region	Number of Cases	Sex (Predominant)	Age (Years)	Age Group	Etiological Agents (%)	Diagnostic Method	References
North America	Canada	Montreal	315	Male	0 to 18	Infant to Adolescent	*M. audouinii* (47.0); *T. soudanense* (38.4); *T. violaceum* (14.3) *T. schoenleinii* (0.3)	Direct microscopy	[9]
		Toronto	107	Female	1 to 12	Child and Adolescent	*M. canis* (100%)	Direct microscopy + Culture	[10]
		Toronto	161	NA	6 to 12	Child and Adolescent	*T. tonsurans* and *T. violaceum* (% NA)	Direct microscopy + Culture	[11]
		Toronto	1	Female	85	Senior elderly	*T. rubrum* (100)	Direct microscopy + Culture	[12]
	USA	Baltimore	24	Male	1 to 47	Adult	*T. violaceum* (66.7); *T. soudanense* (33.3)		[13]
		Birmingham	36	Both	9 to 10	Child and Adolescent	*T. tonsurans* (100)	Direct microscopy + Culture	[14]
		California	495,539	Male	0 to 15	Infant to Adolescent	*T. tonsurans* (91.8); *M. canis* (3.0); NA (5.2)	Direct microscopy + Culture	[15]
		California	1	Male	19	Adolescent	*T. tonsurans* (100)	Direct microscopy + Culture	[16]
		Chicago	1	Male	6	Child	*T. soudanense* (100)	Direct microscopy + Culture	[17]
		Cleveland	122	Male	3 to 12	Child and Adolescent	*T. tonsurans* (99.2); *M. canis* (0.8)	Direct microscopy + Culture	[18]
		Columbus	NA	Female	6 to 13	Child and Adolescent	*T. tonsurans* (88.9); *T. violaceum* (4.2); *M. canis* (2.1); *T. rubrum* (2.1); *T. soudanense* (1.6); *T.verrucosum* (0.5); *T. mentagrophytes* (0.5)	Direct microscopy + Culture	[19]
		Kansas	40	Male	2 to 7	Child	*T. tonsurans* (97.6) *T. rubrum* (2.4)	Direct microscopy + Culture	[20]
		Kansas	689	Male	6 to 10	Child	*T. tonsurans* (100.0)	NA	[21]
		Kansas	1	Male	5	Child	*T. rubrum*	Direct microscopy + Culture	[22]
		Ohio	2	Female	5 and 6	Child	*T. soudanense*	Direct microscopy + Culture	[23]
	Mexico	Guadalajara	7	Male	0 to 11	Infant to Adolescent	*M. canis* (28.6); *T. tonsurans* (28.6); NA (42.8)	Direct microscopy + Culture	[24]
		Guadalajara	913	Male	0 to 9	Infant and Child	*M. canis* (100.0)	Direct microscopy + Culture	[25]
		Jalisco	1	Female	67	Elderly	*T. tonsurans*	Direct microscopy + Culture	[26]
		Jalisco	23	Female	6 to 9	Child	*N. gypsea* (100.0)	Direct microscopy + Culture	[27]
		Mexico City	21	Female	0 to 60	Infant to Adult	*M. canis* (100.0)	Direct microscopy	[28]
		Mexico City	1	Male	6	Child	*M. canis* (100)	Direct microscopy + Culture	[29]
		Michoacan	1	Male	8	Child	*N. nana* (100)	Direct microscopy + Culture	[30]
		Monterrey	441	Both	NA	NA	*T. rubrum* (45), *T. tonsurans* (21), *M. canis* (7.1) (global)	Direct microscopy + Culture	[31]
		Monterrey	1	Male	17	Adolescent	*M. canis* (100)	Direct microscopy + Culture	[32]
		Yucatán	31	Female	1 to 22	Child and young adult	*T. mentagrophytes* (100)	Direct microscopy + Culture	[33]
		Zapopan	1	Female	54	Adult	*T. tonsurans* (100)	Direct microscopy + Culture	[34]
Central America and the Caribbean	Guatemala	Guatemala	325	Male	0 to 72	Elderly	*M. canis* (82.0); *M. gypseum* (6.0) *T. rubrum* (5.0); *T. tonsurans* (2.5); Unidentified (4.5)	Direct microscopy + Culture	[35]
		Guatemala	60	Female	1 to 14	Child and Adolescent	*M. canis* (69.8); *T*. *rubrum* (13.2); *M. gypseum* (13.2); *T. mentagrophytes* (1.9); *M. canis* and *T. rubrum* (1.9)	Direct microscopy + Culture	[36]
		Guatemala	100	Female	1 to 14	Child and Adolescent	*T. rubrum*; *M. canis*; *T. mentagrophytes*; *M. gypseum*; *M. Audouinii* (% NA)	Direct microscopy + Culture	[37]
	Costa Rica	Guanacaste and Puntarenas	2	Both	4 and 5	Child	*T. mentagrophytes* (100)	Direct microscopy + Culture	[38]
		La Carpio-Leon XIII San José	1	Male	9	Child	*M. gypseum* (100)	Direct microscopy + Culture	[1]
	Cuba	La Habana	1	Male	4	Child	*M. canis* (100)	Direct microscopy + Culture	[39]
	Dominican Republic	Santo Domingo and Mexico City	135	Male	5 to 9	Child	*M. canis* (68), *T. tonsurans* (20), *M. audouinii* (9), *T. mentagrophytes* (3)	Direct microscopy + Culture	[40]
		Santo Domingo	37	Male	1 to 9	Child	*T. tonsurans* (41), *M. canis* (16), *M. gypseum* (5); NA (38)	Direct microscopy + Culture	[41]
		Santo Domingo	118	Male	6 to 8	Child	*T. tonsurans* (61), *M. audouinii* (24), *M. canis* (11)	Direct microscopy + Culture	[42]
	Haiti	Puerto Principe	64	Male	1 to 16	Child and Adolescent	*T. tonsurans* (63.6); *T. mentagrophytes* (14.5); *M. audouinii* (12.7); *T. rubrum* (7.3); *M. gypseum* (1.8)	Direct microscopy + Culture	[43]
	Jamaica	Kingston	79	Male	2 to 49	Child to adult	*T. tonsurans* (43.9); *M. audouinii* (37.8); *T. mentagrophytes* (8.5); *T. rubrum* (2.4); *M. canis* (1.2); *M. gypseum* (1.2); NS (4.9)	Direct microscopy + Culture	[44]
South America	Argentina	Tucuman	373	Male	0 to 14	Infant to Adolescent	*M. canis* (93.8); *T. tonsurans* (3.5); *M. gypseum* (1.9); *T. mentagrophytes* (0.8)	Direct microscopy + Culture	[45]
		Buenos Aires	114	Male	5	Child	*M. canis* (61.3); *T. mentagrophytes* (26.1); *T. tonsurans* (12.6)	Direct microscopy + Culture	[46]
		Buenos Aires	269	Male	0 to 49	Infant to Adult	*M. canis* (81.4); *T. mentagrophytes* (8.9); *M. gypseum* (4.5); *T. tonsurans* (2.2); *T. rubrum* (2.2); NA (0.7)	Culture	[47]
		Buenos Aires	1	Female	19	Adolescent	*M. canis*	Direct microscopy + Culture	[48]
		Buenos Aires	1	Female	8	Child	*T. tonsurans*	Direct microscopy + Culture	[49]
		Buenos Aires	1	Female	43	Adult	*M. canis*	Direct microscopy + Culture	[50]
		Buenos Aires	29	Male	3 to 14	Child and Adolescent	*T. tonsurans*	Direct microscopy + Culture	[51]
		Multicenter	439	Female	1 to 11	Child and Adolescent	*M. canis* (66.67), *T. tonsurans* (8.34), *M. gypseum* (8.34)	Direct microscopy + Culture	[5]
	Brazil	Río de Janeiro	78	Male	2 to 12	Child and Adolescent	*M. canis* (59), *T. tonsurans* (24), Other (<5%)	Direct microscopy + Culture	[52]
		Río de Janeiro	6	Male	1 to 9	Child	*M. canis* (66.7), *T. tonsurans* (33.3)	Direct microscopy + Culture	[53]
		Fortaleza	438	Female	0 to 75	Infant to Senior	*T. tonsurans* (54.4); *M. canis* (39.0); *T. rubrum* (4.4); *T. mentagrophytes* (1.5); *M. gypseum* (0.7)	Direct microscopy + Culture	[54]
		Mato Grosso	24	NA	0 to 12	Infant to Adolescent	*M. canis* (53.6), *T. mentagrophytes* (24.3), NA (22.1)	Direct microscopy + Culture	[55]
		Amazonas	153	Male	1 to 9	Child	*T. tonsurans* (~79.0), NA (21.0)	Direct microscopy + Culture	[56]
		Brasilia	1	Female	5	Child	*T. tonsurans* (100.0)	Direct microscopy + Culture	[57]
		Espiritu Santo	11	Male	2 to 6	Child	*M. canis* (100.0)	Direct microscopy + Culture	[58]
		Sao Paulo	30	Male	1 to 9	Child	*M. canis* (56.6), T. *tonsurans* (36.6), Other (<5.0)	Direct microscopy + Culture	[59]
		Sao Paulo	1	Male	5	Child	*M. canis* (100.0)	Direct microscopy + Culture	[60]
		Sao Paulo	99	Male	1 to 15	Child to adolescent	*Trichophyton* spp. (43.43); *M. canis* (37.37) *N. gypseum* (37.37); NA (19.19)	Direct microscopy + Culture	[61]
		Sao Paulo	11	Male	1 to 50	Child to Adult	*M canis* (33.3); *T. mentagrophytes* (33.3); *T. tonsurans* (16.7); *M. gypseum* (16.7)	Direct microscopy + Culture	[62]
		Sao Paulo	23	Female	0 to 20	Infant to young adult	*M. gypseum* (63.6); *M. canis* (9.1); *T. tonsurans* (27.3)	Direct microscopy + Culture	[63]
		Sao Paulo	180	Both	NS	NS	*M. canis* (65); *T. tonsurans* (28.3); Other (<5)	Direct microscopy + Culture	[64]
		Sao Paulo	1	Female	7	Child	*T. mentagrophytes* (100)	Direct microscopy + Culture + biopsy	[65]
		Sao Paulo	112	Male	0 to 15	Infant to Adolescent	*M. canis* (70.53); *T. tonsurans* (23.21); *T. mentagrophytes* (3.57); *M. gypseum* (1.78); *T. rubrum* (0.89)	Direct microscopy + Culture	[66]
		Sao Paulo	1	Male	24	Young adult	*M. canis* (100)	Direct microscopy + Culture	[67]
		Sao Paulo	25	Male	0 to 12	Infant to Adolescent	*T. tonsurans* (60); *M. canis* (40)	Direct microscopy + Culture	[68]
		Sao Paulo	596	Male	0 to 20	Infant to young adult	*M. canis* (82.2); *T. tonsurans* (4.7); *T. rubrum* (3.3); *N. gypsea* (1.9); *T. mentagrophytes* (1.6); *Trichophyton* spp. (0.8)	Direct microscopy + Culture	[69]
		Goiânia	164	Male	0 to 3	Infant and Child	*M. canis* (71.3); *T. tonsurans* (11) *T. mentagrophytes* (7.9)	Direct micros- copy + Culture	[70]
		Paraíba	53	Female	0 to 60	Infant to elderly	*T. rubrum* (37.7); *T. tonsurans* (28.3); *M. canis* (24.5); *T.mentagrophyes* (9.5)	Direct microscopy + Culture	[71]
		Santa Catarina	1	Female	65	Elderly	*T. violaceum* (100)	Direct microscopy + Culture	[72]
	Bolivia	La Paz	7	Female	1 to 8	Child	*T. mentagrophytes* (33.3); *M. gypseum* (33.3); *T. rubrum* (16.7); *M. canis* (16.7)	Direct microscopy + Culture	[73]
	Chile	Valparaiso	24	Female	1 to 8	Child	*M. canis* (92.9); *M. audouinii* (7.1)	Direct microscopy + Culture	[4]
		Santiago	1	Male	11	Adolescent	*M. canis* (100)	Direct microscopy + Culture	[74]
		Santiago	73	Male	1 to 9	Child	*M. canis* (86.6); *T. tonsurans * (3.3), *M. gypseum* (1.6), *T. rubrum* (1.6); NA (6.9)	Direct microscopy + Culture	[75]
		Santiago	155	Male	1 to 9	Child	*M. canis * (89.8), *T. tonsurans* (7.1) NA (3.1)	Direct micros- copy + Culture	[76]
		Valparaíso	166	NS	1 to 8	Child	*M. canis* (100)	Direct microscopy + Culture	[77]
		Santiago	155	Male	1 to 14	Child and Adolescent	*M. canis* (89.8); *T. tonsurans* (7.1); *T. rubrum* (1.9); *M. gypseum* (0.6); NA (0.6)	Direct microscopy	[77]
	Colombia	Medellín	133	Male	1 to 10	Child and Adolescent	*M. canis* (86.0); *M. gypseum* (4.0); *T. tonsurans* (3.0); *M. adouinii* (3.0); *T. mentagrophytes* (3.0); NS (1.0)	Direct microscopy + Culture	[78]
		Cauca	32	Male	1 to 9	Child	*T. tonsurans* (63); NA (37)	Direct microscopy + Culture	[79]
		Medellín	15	NS	0 to 58	Infant to Adult	*M. canis* (86); Other (<5)	Direct microscopy + Culture	[80]
	Ecuador	Azuay and Cuenca	17	Male	3 to 14	Child and Adolescent	*T. mentagrophytes* (27.7) *T. tonsurans* (22.2); *M. audouinii* (16.7); *T. concentricum* (5.6); *T. violaceum* (5.6); NA (22.2)	Direct microscopy + Culture	[81]
	French Guiana	Cayena	202	Female	1 to 87	Child to Senior	*T. tonsurans* (73.9); *T. mentagrophytes* (8.4); *M. canis* (7.5); *M. audouinii* (5.0); *M. gypseum* (1.7); *M. langeronii* (0.8); *T. rubrum* (0.8); *T. verrucosum* (0.8); NA (1.1)	Direct microscopy	[82]
	Paraguay	Asunción	132	Male	0 to 14	Child to adolescent	*M. canis* (45.4); *T. mentagrophytes* (47.0); *T. rubrum* (3.0); NA (4.6)	Direct microscopy + Culture	[83]
		Asunción	1	Female	6	Child	*M. canis* (100)	Direct microscopy + Culture	[84]
		Asunción	436	Female	0 to 57	Infant to Adult	*M. canis* (52.1); *T. mentagrophytes* (17.0); *T. tonsurans* (16.5); *T. rubrum* (8.7); *M. gypseum* (4.3); *T. verrucosum* (1.1); *M. nanum* (0.2)	Direct microscopy + Culture	[85]
		Itauguá	54	Female	0 to 12	Infant to Adolescent	*T. mentagrophytes* (32.3); *T. rubrum* (29.0); *M. canis* (25.8); *T. tonsurans * (3.2); *M. gypseum* (3.2); NA (6.4)	Direct microscopy	[86]
	Peru	La Libertad	78	Female	0 to 13	Infant to Adolescent	*M. canis* (87.5); *T. mentagrophytes* (3.1); *T. rubrum* (3.1); NA (6.3)	Direct microscopy + Culture	[88]
		Lima	50	Female	1 to 14	Child and Adolescent	*T. tonsurans* 74%, *M. canis* 26%	Direct microscopy + Culture	[88]
	Uruguay	Montevideo	22	Female	0 to 11	Infant to Adolescent	*M. canis* (77.8); *M. gypseum* (11.1); *T. verrucosum* (11.1)	Direct microscopy	[47]
	Venezuela	Anzoátegui	1317	Female	0 to 15	Infant to Adolescent	*M. canis* (<100)	Direct microscopy + Culture	[2]
		Carabobo	12	Male	3 to 8	Child	*M. canis* (100)	Direct microscopy	[89]
		Caracas	71	NA	0 to 9	Infant and Child	*M. canis* (87.7%), *T. tonsurans* complex (9.1%); *T. mentagrophytes* Complex (1.8%); *T. rubrum* (1.4%)	Direct microscopy + Culture	[90]
		Caracas	296	Male	0 to 15	Infant to Adolescent	*M. canis* (82.1); *T. tonsurans* (17.9)	Direct microscopy + Culture	[91]

Note: NA = Not available.

## Data Availability

The data supporting the findings of this study are available within the article and its Supplementary Materials, including the PRISMA flow diagram, study characteristics table, and risk of bias assessment. The data extraction forms and additional materials generated during the systematic review process are available from the corresponding authors upon reasonable request.

## Supplementary Materials

The following supporting information can be downloaded at: https://www.mdpi.com/article/10.3390/biology15161413/s1, Supplementary Table S1. Detailed Search Strategies Used in Each Database; Supplementary File S1: biology-15-01413-PRISMA_2020_checklist PAG
