# Supplementary material for: Tinea capitis in the Americas: Epidemiological Trends and Etiological Distribution from a Systematic Review
Source: Biology (Basel). 2026 Aug 17;15(16):1413. doi: 10.3390/biology15161413 (PMC13510186; doi:10.3390/biology15161413)
Supplement: Supplementary file 1 [file biology-15-01413-s001.zip › biology-4403877-Supplementary Table S1.pdf]

Supplementary Table S1. Detailed Search Strategies Used in Each Database

Database	Search strategy
PubMed	("tinea capitis" OR dermatophyte* OR Trichophyton OR Microsporum OR Nannizzia) AND ("North America" OR "Central America" OR "South America" OR Caribbean OR country names)
Scopus	Same terms adapted to database syntax
SCILIT	Same terms adapted to database syntax
SciELO	Same terms adapted to database syntax
